# Effect of PEG grafting density on surface properties of polyurethane substrata and the viability of osteoblast and fibroblast cells

**DOI:** 10.1007/s10856-022-06668-1

**Published:** 2022-05-18

**Authors:** A. D. Abreu-Rejón, W. Herrera-Kao, A. May-Pat, A. Ávila-Ortega, N. Rodríguez-Fuentes, J. A. Uribe-Calderón, J. M. Cervantes-Uc

**Affiliations:** 1 A.C, Unidad de Materiales, Calle 43 No. 130, Centro de Investigación Científica de Yucatán, Col. Chuburná de Hidalgo, C.P. 97205 Mérida, Yucatán México; 2 Facultad de Ingeniería Química, Periférico Norte Km 33.5 Tablaje Catastral 13615, Universidad Autónoma de Yucatán, Chuburná de Hidalgo Inn, C.P. 97203 Mérida, Yucatán México; 3 Centro de Investigación Científica de Yucatán, A.C, Unidad de Materiales, Calle 43 No. 130, CONACYT, Col. Chuburná de Hidalgo, C.P. 97205 Mérida, Yucatán México

## Abstract

The surface of Tecoflex SG-80A Polyurethane (PU) films was modified by grafting polyethylene glycol (PEG) chains at three different molar amounts (0.05, 0.10, and 0.15 mmol). The resulting substrata were characterized by FTIR-ATR, TGA, AFM, SEM and contact angle to assess the surface modifications occurred during the grafting reactions. Osteoblasts and fibroblasts were cultured with PU extracts for 24 h, and their cell viability and morphology were evaluated by CellTiterBlue assay, Crystal Violet staining and Live/Dead assay. FTIR and TGA results indicated that PEG chains were successfully grafted onto PU surfaces, specifically in the hard segment of PU forming allophanate groups as the PEG grafting density increased. SEM and AFM images suggest that PU substrata were partially covered by PEG, increasing the dispersive and basic components of the PU surface energy. It was found that extracts from PEG-grafted polyurethanes increased the osteoblast viability, although fibroblasts viability remained constant regardless PEG grafting density; in spite of this both cells presented a more spread morphology at the lower PEG grafting density. Our results showed that surface energy of PU substrata can be tuned by PEG grafting density; also, the PEG leached tends to increase the pH of culture medium which leads to a higher viability of osteoblasts; nevertheless, PEG grafting density should be optimized to promote a healthy cell morphology as alterations in its morphology were detected at higher concentrations.

**Figure:**
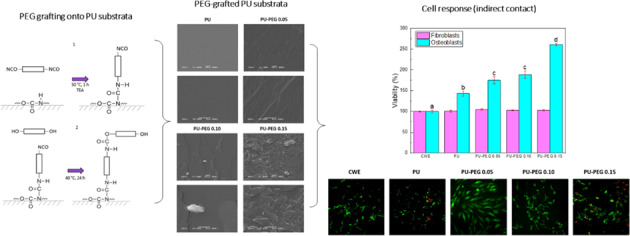
Graphical abstract

Graphical abstract

## Introduction

Polyurethanes (PUs) are widely used for biomedical applications and for tissue engineering due to their good biocompatibility and mechanical properties. In this regard, materials surface characteristics are of great importance as the interactions between them and biologic elements occur at the surface. Cellular adhesion is of particular importance as it mediates cell behavior and survival [1–3]. Most polymers, including PUs, do not promote cell adhesion due to their low hydrophilicity [4, 5], which can produce a strong protein adsorption and denaturation, reducing their biocompatibility and causing thrombosis or foreign body reaction [6–10]. In order to improve cellular adhesion onto polymeric surfaces, chemical or physical modifications have been performed to make them more hydrophilic [11]. A common strategy to make polymeric surfaces more hydrophilic and improve their biocompatibility is to graft polyethylene glycol (PEG) chains onto them [8, 12]. PEG is highly hydrophilic and has good biocompatibility, which makes it a widely used polymer in biomedical devices. These features lead to that the grafting of PEG chains onto the surface of different polymers increase their biocompatibility and cell viability [13–16]. However, PEG grafting can also create inactive surfaces sites that do not promote cell adhesion due to the exclusion effect that creates when water molecules interact with PEG chains [17–20], i.e., the protein adsorption and cell adhesion levels depend on the amount of PEG grafted onto the surface [21]; therefore, by adjusting the grafting density of this polymer, bioactivity of surfaces could be controlled [22–24]. For example, Zhou et al. [25] synthesized a PU-g-PEG copolymer and reported tunable resistance to protein adsorption of PUs by varying PEG grafting density. Then, the surface properties and bioactivity of polymeric substrates can be improved with an appropriated PEG grafting density. Moreover, some works have reported an increased viability and activity of cells when PEG is included in biomaterials [26, 27]. In this regard, Mao et al. [28] synthesized PEGylated trimethyl chitosan copolymers that promoted a higher viability in fibroblasts than those exhibited by respective controls. On the other hand, Su et al. [29] reported an enhanced viability of human endothelial cell for methoxy polyethylene glycol-g-PU compared to controls. Cai et al. [30] reported higher alkaline phosphatase (ALP) activity and cell attachment of MC3T3 osteoblasts on PEG-grafted polypropylene fumarate (PPF) compared to PPF and tissue culture polystyrene (TCPS).

Though there are many works on the use of PEG to improve biocompatibility of polymer surfaces, some of them have investigated the increased viability in some cells exerted by PEG grafting, but this effect is not yet understood. Therefore, the aim of this work was to evaluate the effect of PEG grafting density using different amounts of PEG on the surface properties and cell viability of commercially available PU substrata; for the latter, osteoblasts and fibroblasts were employed.

## Materials and methods

### Materials

Tecoflex^TM^ SG-80A pellets from Lubrizol was used for the preparation of PU substrata. Polyethylene glycol (PEG, Mn 2000), hexamethylene diisocyanate (HMDI, 98%), triethylamine (TEA, 99%) and toluene (99.5%) were purchased from Sigma-Aldrich.

### Polyurethane films preparation

Tecoflex films were elaborated by solvent-casting technique. PU was dissolved in tetrahydrofuran (1:15 w/v) with magnetic stirring for 24 h at room temperature; then the dissolution was poured into Petri dishes and left in an extraction hood for 24 h. Finally, films were dried in a vacuum oven for 24 h at 60 °C.

### Grafting of PEG onto polyurethane substrata

**Fig. 1:**
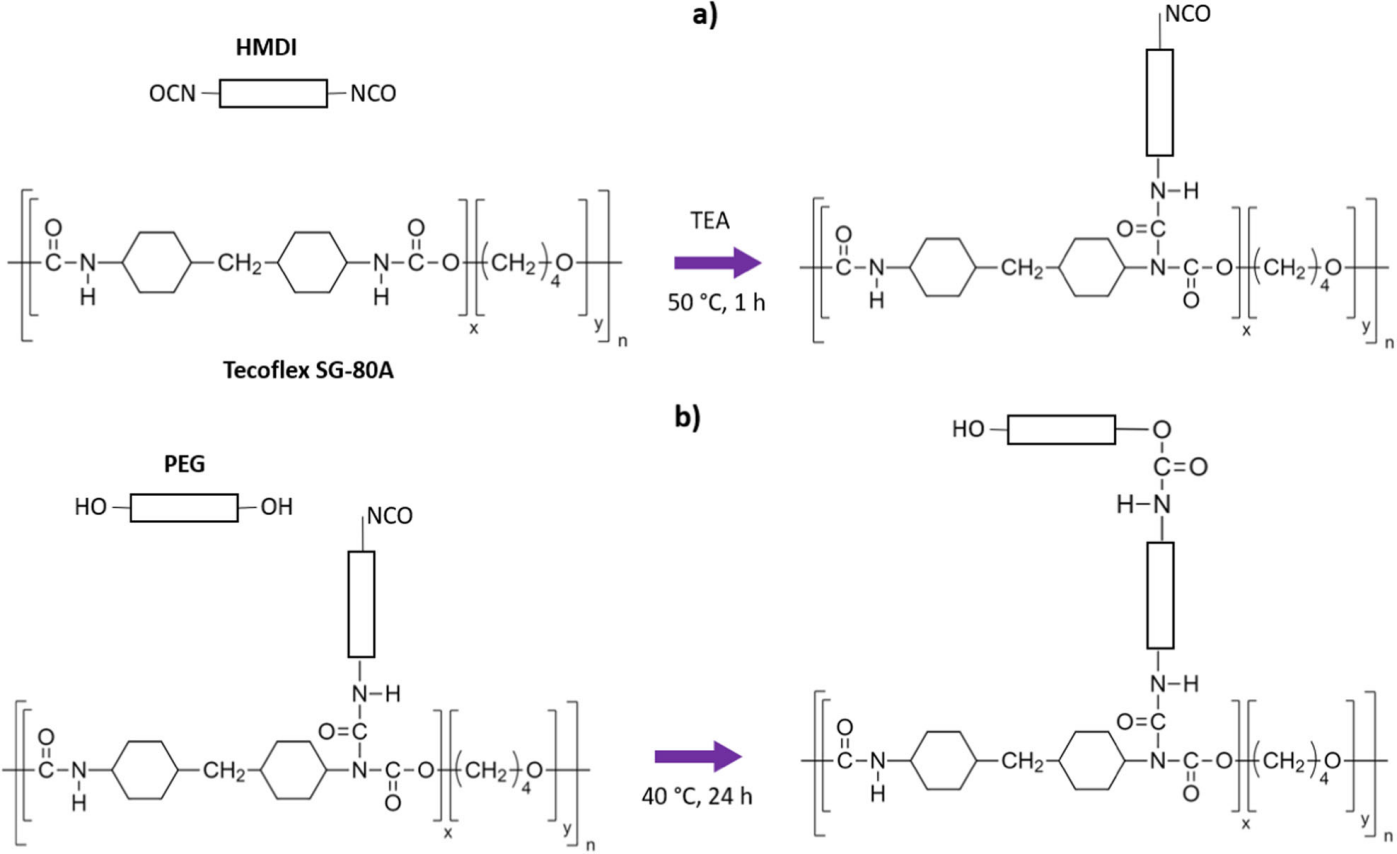
Grafting reaction of PEG onto PU substrata. (**a**) One isocyanate group from end groups of HMDI reacts with the secondary amine of the urethane group to yield allophanate linkages remaining an isocyanate group unreacted (first step); (**b**) hydroxyl group from PEG reacts with the free isocyanate group obtained in the previous stage to yield a new-urethane linkage (second step)

PEG grafting onto PU films was done following the procedure reported by Freij-Larsson and Wesslén [31] which is illustrated in Fig. 1. Briefly, PU films were immersed in HMDI/TEA (3:1 v/v ratio) toluene solution (30 mL) for 1 h at 50 °C, with magnetic stirring under nitrogen atmosphere. After this, films were rinsed with fresh toluene and then immersed in a solution of PEG in toluene at 40 °C for 24 h. Finally, the films were rinsed again in toluene and dried in an extraction hood for 72 h. HMDI and PEG were added at three different equimolar amounts (0.05, 0.10, and 0.15 mmol) per 1 g of PU.

### Physicochemical characterization

#### Contact angle and surface free energy measurements

Contact angles of sessile drops (5 µL) of distilled water, glycerol and diiodomethane were measured in a ramé-hart 250-U1 goniometer. Surface free energy including dispersive, basic and acid components were determined with van Oss and Good model using the following equation [32]:

1 $$\frac{1}{2}\left( {1 + \cos \theta } \right)\gamma _L = \left( {\gamma _S^{LW}\gamma _L^{LW}} \right)^{1/2} + \left( {\gamma _S^ + \gamma _L^ - } \right)^{1/2} + \left( {\gamma _S^ - \gamma _L^ + } \right)^{1/2}$$

where θ represents the contact angle, *γ*_*L*_ is the surface free energy of liquid, $\gamma _L^{LW},\,\gamma _L^ - ,\,\gamma _L^ +$ are the corresponding dispersive, basic and acid components of the surface free energy of the liquid, whereas $\gamma _S^{LW},\,\gamma _S^ - ,\,\gamma _S^ +$ are the dispersive, basic and acid components of the solid surface free energy, respectively.

#### Infrared spectroscopy

Fourier transform infrared (FTIR) spectra of the substrata were obtained with a Thermo Fisher Scientific Nicolet 8700 spectrometer equipped with an attenuated total reflectance (ATR) accessory of ZnSe. The scans were obtained from 4000 to 600 cm^−1^ spectral range with a resolution of 4 cm^−1^ averaging 100 scans.

#### Thermogravimetric analysis

Thermogravimetric analysis (TGA) was performed in a Perkin-Elmer TGA 8000 equipment, at a heating rate of 10 °C/min from 50 to 650 °C under nitrogen atmosphere, the sample mass was 10 mg.

#### Raman spectroscopy

Raman spectroscopy was carried out in a Renishaw inVia Raman spectrometer with a 633 nm laser; the exposure time was 10,000 ms at a 100% power. The scans were performed in the region of 3300–450 cm^−1^.

#### Scanning electron microscopy

Surface topography of the substrata was observed by scanning electron microscopy (SEM) using a JEOL JSM-6360LV microscope with acceleration voltage of 20 kV. Samples were coated with a thin layer of gold and examined at 25 °C.

#### Atomic force microscopy

Surface roughness of modified substrata was characterized by atomic force microscopy (AFM) in a Bruker INNOVA microscope equipped with a Bruker TESP nanoprobe silicone tip (spring constant of 42/Nm and 2 nm tip radius). Images were obtained at room temperature in tapping mode with a resonant frequency of 320 kHz and a scanning frequency of 0.3 Hz. The scanning area of 20 µm × 20 µm was divided into four sub-areas of 10 µm × 10 µm; then, the roughness of each sub-area was obtained with the Nanoscope Analysis software. Statistic average and standard deviation was reported for each specimen.

### Cellular studies

#### Cell culture

Human fibroblasts and murine osteoblasts were grown in T-25 cell culture flasks with cell culture medium Dulbecco’s Modified Eagle’s Medium (DMEM) supplemented with 10% fetal bovine serum, 1% antibiotics (streptomycin 100 µg/mL, penicillin G sodium 100 U/mL) at 37 °C and 5% CO_2_; 50 µg/mL of ascorbic acid was added to osteoblast medium. Cells were detached at 80% confluence with a solution of 0.25% trypsin-EDTA and incubated for 4 min under standard conditions; this suspension was added to cell culture medium (1:1) and centrifuged for 10 min at 1200 rpm in a Centurion Scientific Limited K241 centrifuge; then supernatant was separated and cells resuspended in 1 mL of fresh culture medium. Viable cells were counted using a mixture of trypan blue and cell suspension (1:1) in a hemocytometer (Neubauer cell chamber).

#### Viability study

Cell viability was evaluated by indirect test from extracts of the materials under standardized conditions (ISO 10993-5). The films, previously sterilized by UV radiation and washed with phosphate-buffered saline (PBS), were immersed in culture medium in a 100:2.5 (mg/mL) ratio, and incubated under standard culture conditions for 3 days. Osteoblasts and fibroblasts were seeded in 96-well plates at a density of 5 × 10^3^ cells per well with 100 µL of cell culture medium and incubated for 24 h; these cells were selected to study the effect of PEG grafting on the viability of cells from a hard tissue (osteoblasts) and a soft tissue (fibroblasts). Then, the culture medium was withdrawn and 100 µL of the extracts was added to the wells, and incubated for another 24 h; cell culture medium and hydrogen peroxide were used as positive and negative controls, respectively. Afterward, CellTiterBlue was added to the samples (20% v/v) and incubated for 4 h. The absorbance was measured in a Cytation 3 plate reader (BioTek) at 570 nm. Cell viability was calculated using the following equation:

2 $$Cell\,viability\,\left( {{{\mathrm{\% }}}} \right) \,=\, \frac{{A \,-\, A_n}}{{A_p \,-\, A_n}}\,\times\,100$$

where A is the absorbance of the test well and, A_p_ and A_n_ are the absorbance of the positive and negative controls respectively.

#### Crystal violet staining

Morphology of the cells was assessed by inverted field microscopy after crystal violet staining of the remaining cells of the proliferation assay. Culture medium was withdrawn from the test wells and rinsed twice with PBS; then cells were fixed with 50 µL of methanol and incubated for 5 min. Afterward, methanol was withdrawn and the remaining dried for 30 min. 50 µL of crystal violet solution was added to the wells and incubated for 5 min; finally, the cells were rinsed with distilled water and observed in a Labomed TCM 400 microscope.

#### Live/dead assay

Live/Dead assay was performed to investigate the viability of cells. Osteoblasts were cultured on coverslips in a 48-well plate for 3 days; then, these cells were incubated for 24 h with 200 µL of the extracts from the PU substrata. After this, the extracts were withdrawn and 100 µl of the assay solution [3 µmol calcein and 3 µmol of ethidium homodimer-1 (EthD-1) in 1 mL PBS] was placed in the wells for 1 h at 37 °C in the dark. Later, the coverslips were placed on slides with 10 µl of mounting medium. Samples were analyzed with an Olympus Corp. FV-100 microscope and the fluorescence was observed at 421 nm for calcein and 480 nm for EthD-1.

#### Statistical analysis

All data were analyzed by one-way ANOVA, followed by Tukey’s multiple comparison tests using the software Origin (2008) with significance of *P* < 0.05.

## Results

### Spectroscopic analysis

**Fig. 2:**
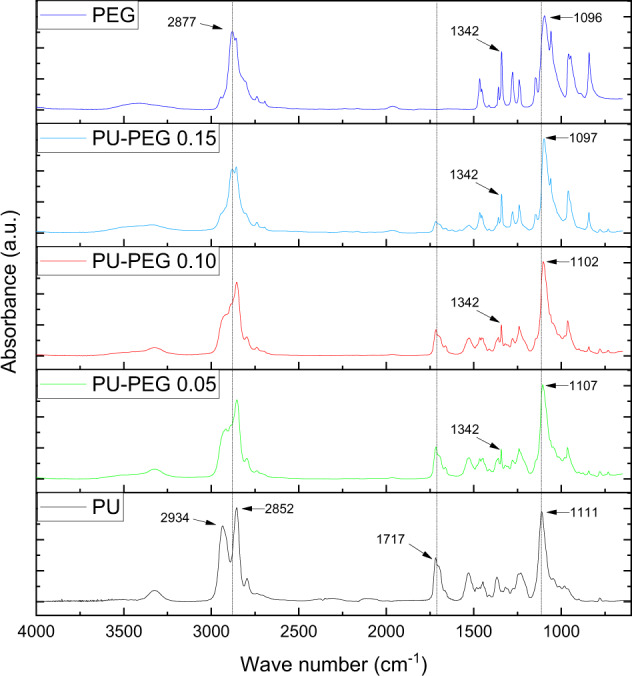
FTIR-ATR spectra of the PU films and PEG. As the graft density is increased the spectra of the films look more similar to PEG spectra, indicating that there is a higher amount of PEG chains present

Figure 2 shows the FTIR spectra of the unmodified PU, PEG and, PEG-grafted PU samples. Spectrum of unmodified PU shows bands at 2934 and 2852 cm^−1^, which corresponds to asymmetric and symmetric stretching vibration of C–H linkage; a band at 1717 cm^−1^ due to C=O stretching vibration (amide I), and a band at 1111 cm^−1^ associated with C–O stretching. For its part, the FTIR spectrum of PEG exhibits bands at 2877 cm^−1^ of C–H stretching vibration; 1342 cm^−1^ for O–H bending, and the band of C–O–C stretching vibration at 1096 cm^−1^. Interestingly, the spectra of the grafted PU films are similar to that exhibited by PEG, being more similar to PEG spectrum as the PEG amount increases. Thus, the bands located at 2934 and 1717 cm^−1^ of unmodified PU spectrum tend to disappear in PEG-grafted PU spectra while, the band at 2852 cm^−1^ shifts to higher wave numbers, close to the values displayed by PEG spectrum. Similarly, the bands related to C–O bond at 1111 cm^−1^ of modified PUs shift to lower wave numbers up to 1097 cm^−1^ for modified substrata. Additionally, spectra of treated substrata show the appearance of a band at 1342 cm^−1^ associated with O–H bending vibration which is not present in the unmodified PU.

The reduction of the characteristic bands belonging to PU in the FTIR spectra as the PEG amount increases can be monitored by calculating the bands ratio 1717 cm^−1^/~1100 cm^−1^ (the first bands is associated to carbonyl band of urethane groups, while the second one is related to C–O linkage from PU and PEG). As can be seen, the ratio decreases from 0.47 for unmodified PU to 0.34, 0.28, and 0.12 for PU-PEG 0.05, PU-PEG 0.10, and PU-PEG 0.15, respectively.

**Fig. 3:**
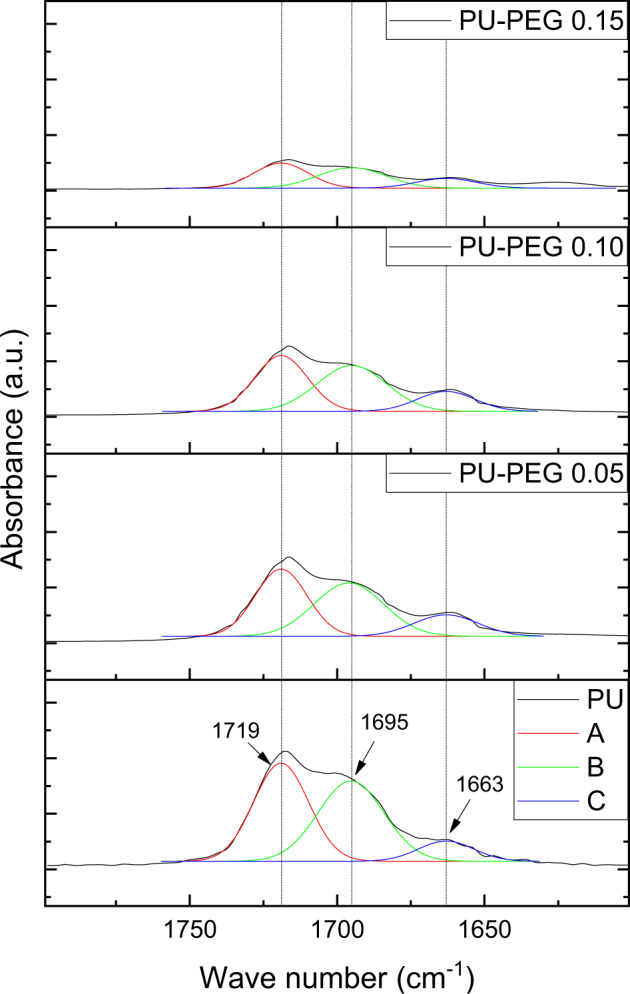
Deconvolution of carbonyl bands from 1800 to 1600 cm^–1^. The signals at 1719 cm^−1^ (**A**) are assigned to non-hydrogen-bonded carbonyls, 1695 cm^−1^ (**B**) to hydrogen-bonded carbonyls, and 1663 cm^−1^ (**C**) to carbonyls from allophanate groups

Since PEG grafting onto PU substrata yields allophanate groups during the first stage of reaction and a new-urethane linkage during the second one, a deconvolution analysis of the carbonyl bands was performed to analyze the changes in the urethane linkage environment, and results are shown in Fig. 3. It can be seen that the carbonyl band is formed by the contribution of three signals. The first signal (A), at 1719 cm^−1^, is related to the stretching vibration of the free carbonyl from the urethane group whereas the second one (B), at 1695 cm^−1^, is associated with the hydrogen-bonded carbonyl of the same group [33]. Finally, the third signal located at 1663 cm^−1^ (C) has been related to the carbonyl from the urea-like substructure of allophanate groups [34] which appears in PU spectra due to side reactions of the isocyanate moiety. Interestingly, the intensity ratio of bands C/(A + B) increases as grafting concentration of the PU films increases (see Table 1); which indicates that the number of allophanate groups increases with respect to the urethane bonds. The ratio A/B also increases for all the treated films, as there exist more non-hydrogen-bonded carbonyls, in comparison to that exhibited by unmodified PU.

**Table 1:** Carbonyl FTIR band ratios

	A/B	C/(A + B)
PU	1.18	0.15
PU-PEG 0.05	1.23	0.21
PU-PEG 0.10	1.19	0.23
PU-PEG 0.15	1.20	0.24

**Fig. 4:**
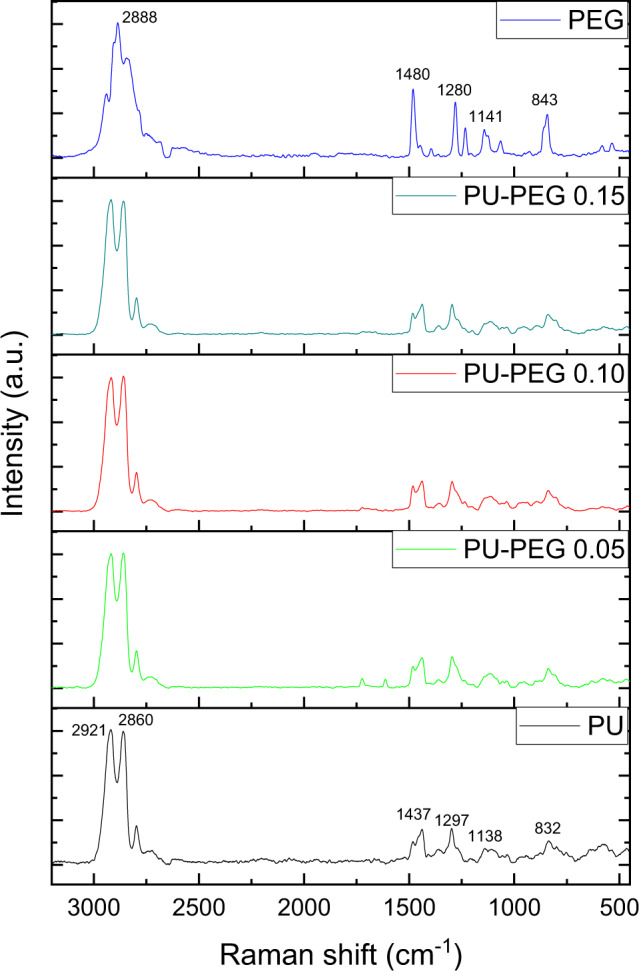
Raman spectra of the PU films and PEG

The Raman spectra of the unmodified and modified-PU substrata, as well as PEG are shown in Fig. 4. As noted, PU exhibits two very strong bands at 2921 and 2860 cm^−1^ corresponding to the asymmetric and symmetric stretching vibration of the C–H linkage, respectively; a band at 1437 cm^−1^ due to CH_2_ scissoring vibration, other band at 1297 cm^−1^ associated with C–C chain vibrations, and the bands at 1138 cm^−1^ and 832 cm^−1^ assigned to the asymmetric and symmetric stretching vibrations of the C–O–C group were also observed. The Raman spectra of PEG shows a very strong band at 2888 cm^−1^ for the stretching vibration of the C–H linkage. The band at 1480 cm^−1^ is assigned to the bending vibration of CH_2_. The bands at 1280 and 1232 cm^−1^ correspond to the C–C stretching vibration. Finally, the bands at 1141 and 843 cm^−1^ are due to the C–O–C asymmetric and symmetric vibrations, respectively. In contrast with the changes displayed by FTIR spectroscopy, Raman spectra did not show significant changes between unmodified and modified-PU substrata.

### Thermal properties

TGA and DTGA curves of unmodified and modified PUs as well as PEG can be seen in Online Resource 1 and 2. Unmodified PU exhibits two mass losses; the first weight loss located at 247 °C, is associated with the degradation of the hard segment of segmented polyurethane by the cleavage of the urethane bond, accounting for the 28% of the total mass. The second one at 413 °C is related to the decomposition of the soft segment [35]. In contrast, PEG thermograms exhibit only one degradation stage at 405 °C.

It is also observed that thermal stability of the PU films is reduced with the grafting of PEG. Thus, the onset degradation temperature is lowered from 270 to 200 °C, as can be clearly noted in DTGA curves. Here, results show how the hard segment degradation of these films starts at lower temperatures compared to unmodified PU. The Td_max_ of the hard segment degradation in DTGA shifts to lower temperatures for the treated PU films. Even though the Td_max_ of the hard segment in the grafted substrata is lower than in PU, it increases with the grafting density. In addition, the Td_max_ of the soft segment degradation in the grafted substrates decrease from 413 to 405 °C.

### Topography of the substrata

**Fig. 5:**
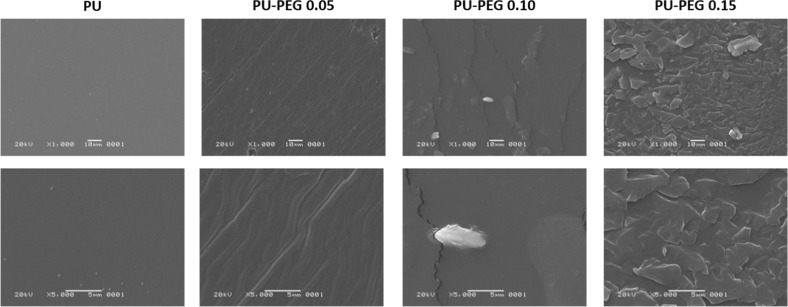
SEM images of the grafted films. A PEG covering is formed on the surface of the substrata

**Fig. 6:**
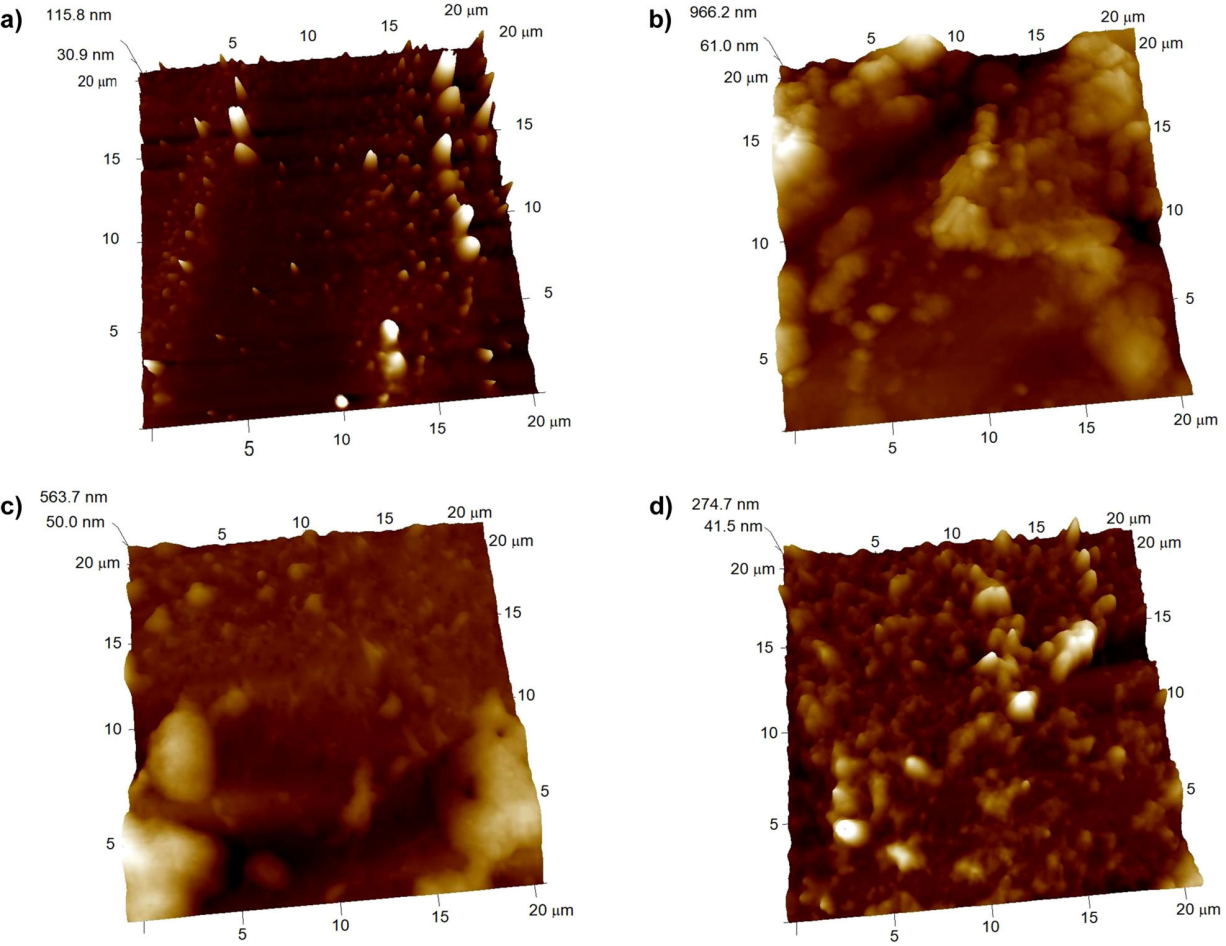
AFM images of untreated PU and PEG-grafted PU films. **a** PU, (**b**) PU-PEG 0.05, (**c**) PU-PEG 0.10, (**d**) PU-PEG 0.15

The SEM images presented in Fig. 5 show the surface of the grafted and non-grafted substrata. It can be seen that surface topography of the substrates is modified by the presence of PEG; i.e., topology changes from a homogeneous one for unmodified PU, to more uneven surfaces as the PEG concentration increased for the grafted PU films. PU-PEG 0.05 films presented grooves along their surface, which can be attributed to a non-uniform PEG covering; this covering seems to become uniform for PU-PEG 0.10 sample, but some ridges can be observed due to the clustering of PEG chains. The number of the ridges increased on the surface of PU-PEG 0.15 films as a consequence of the higher PEG grafting density. AFM images in Fig. 6 also show that the topography of the substrates becomes more irregular by the grafting reaction.

### Contact angles and surface energy

Table 2 summarizes the contact angles of sessile drops of water, glycerol and diiodomethane on the PU films. As expected, the hydrophilicity of the substrata increased with PEG grafting density as shown by the water contact angle values. Interestingly, the contact angle values with glycerol and diiodomethane also decreased.

**Table 2:** Contact angles and surface free energy components of the films as determined by the method of van Oss and Good

	Water (°)	Glycerol (°)	Diiodomethane (°)	Surface energy (mJ/m^2^)	Ys^LW^ (mJ/m^2^)	Ys^ab^ (mJ/m^2^)	Ys^−^ (mJ/m^2^)	Ys^+^ (mJ/m^2^)
PU	71 ± 1^a^	69 ± 2^a^	50 ± 1^a^	36.1 ± 1^a^	34.3 ± 1^a^	1.7 ± 1^a^	16.0 ± 2^a^	0.1 ± 0.01^a^
PU-PEG 0.05	54 ± 2^b^	61 ± 2^b^	37 ± 1^b^	43.7 ± 1^b^	41.0 ± 1^b^	2.6 ± 1^b^	31.5 ± 4^b^	0.1 ± 0.01^a^
PU-PEG 0.10	53 ± 3^b^	55 ± 2^c^	27 ± 1^c^	47.1 ± 1^c^	45.2 ± 1^c^	1.8 ± 1^b^	27.2 ± 5^b^	0.1 ± 0.01^a^
PU-PEG 0.15	39 ± 1^c^	47 ± 4^d^	27 ± 3^c^	50.1 ± 3^c^	45.4 ± 1^c^	4.7 ± 2^c^	39.9 ± 4^c^	0.2 ± 0.01^b^

Equal letters denote that the difference is not statistically significant

*Ys*^*LW*^ Non-polar component, *Ys*^*ab*^ Polar component, *Ys*^*−*^ Basic component, *Ys*^*+*^ Acid component

Surface energy of substrates was calculated as the sum of dispersive (Ys^LW^) and polar (Ys^ab^) components, which were determined from contact angles measurements; these values are also listed in Table 2. As noted, the surface energy values of the grafted PU films increased with the grafting density, mainly by the increment of the dispersive component. In general, the polar component for PU-PEG 0.05 and PU-PEG 0.10 did not change, but it increased for PU-PEG 0.15. Results also shown that there was an improvement in the basic component (Ys^−^) of the grafted films; however, a slight increment in the acid component (Ys^+^) can be also noted in PU-PEG 0.15. All these changes showed statistical difference with *P* < 0.05.

### Cellular response to the extracts of the films

**Fig. 7:**
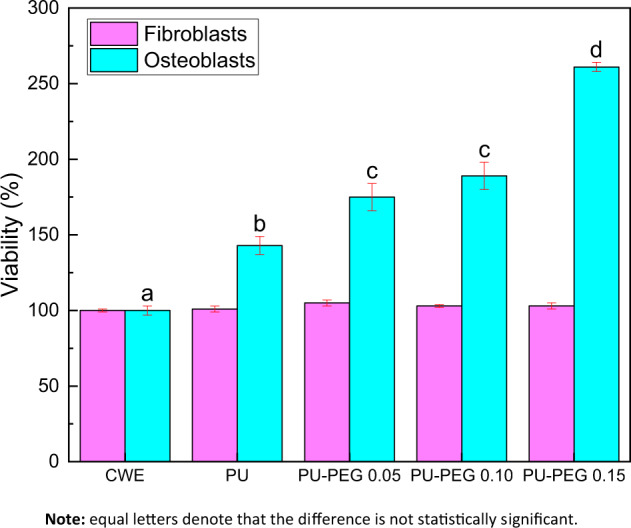
Cell viability of osteoblast and fibroblasts cells by indirect contact with PU substrata for 24 h. CWE: cells without contact with extracts of the films. *P* < 0.001, one-way ANOVA with Tukey’s multiple comparison test, *n* = 5 in each group

Figure 7 shows the viability of osteoblasts and fibroblasts in contact with the extracts of the substrata. None of the extracts of the materials presented a viability lower than the cell culture without exposition to the extracts (CWE), indicating that the leachables from substrata are not cytotoxic. Osteoblasts showed a higher viability than that exhibited by CWE, in all substrata, 140%, 175%, 189%, and 261% for PU, PU-PEG 0.05, PU-PEG 0.10, and PU-PEG 0.15, respectively; grafted PUs also exhibited higher viability respect non-grafted PU. In contrast, fibroblasts viability was not affected by the contact with any extract.

**Fig. 8:**
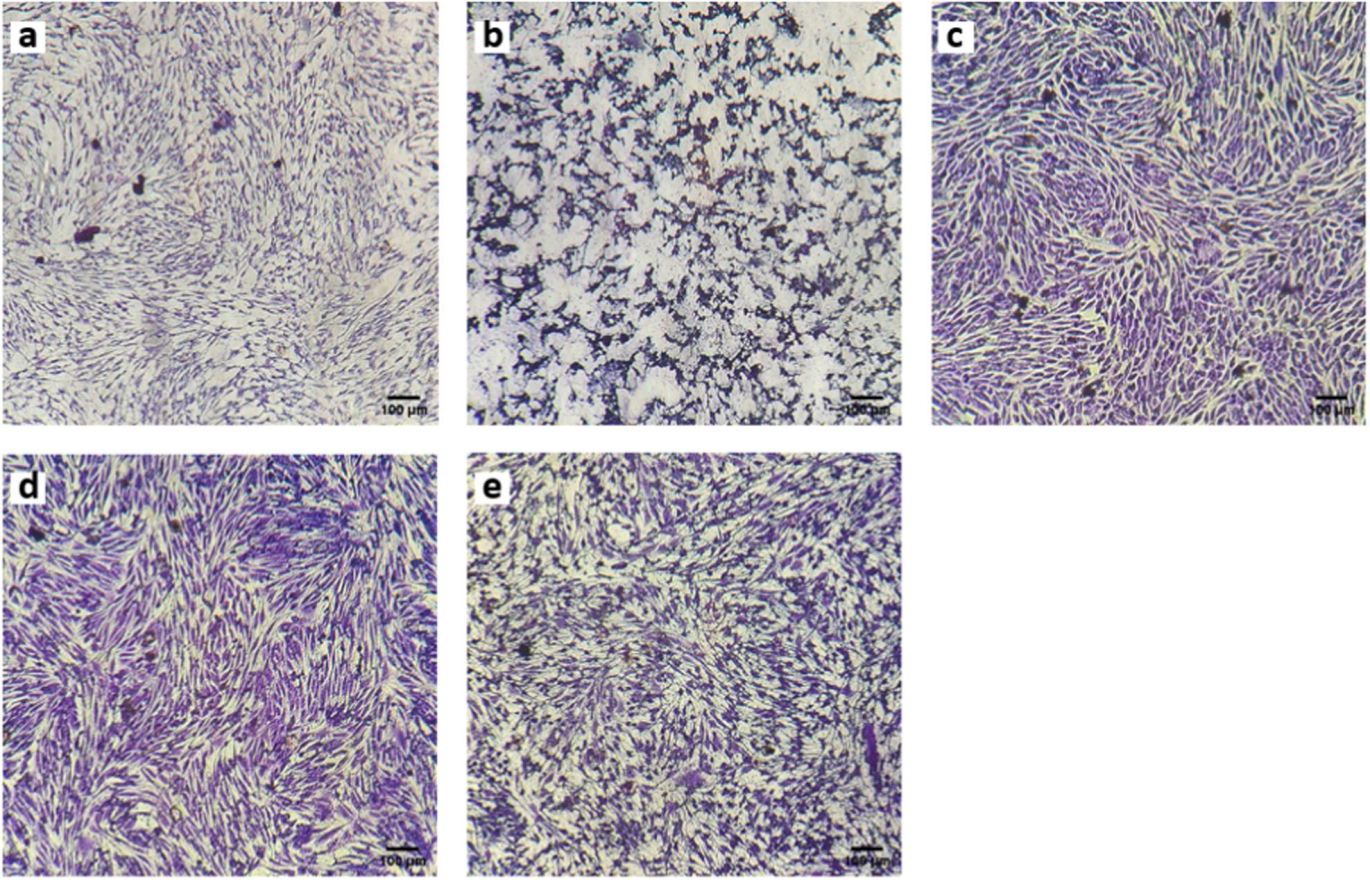
Microscopic images of Crystal Violet-stained osteoblasts in contact with extracts of the films for 24 h. **a** CWE, (**b**) PU, (**c**) PU-PEG 0.05, (**d**) PU-PEG 0.10, (**e**) PU-PEG 0.15

It is interesting to note that the morphology of the cells was affected by the leachables from grafted PUs (see Fig. 8). Osteoblasts in contact with extracts from untreated PU showed an irregular morphology with membrane blebbing, as if they were in apoptosis; however when cells were in contact with the extracts from PU-PEG 0.05 and PU-PEG 0.10, they exhibited an extended and regular morphology of adhered osteoblasts [2], and even orientation across the wells. Interestingly, osteoblast morphology in contact with extracts from PU-PEG 0.15 was again altered showing an irregular form.

**Fig. 9:**
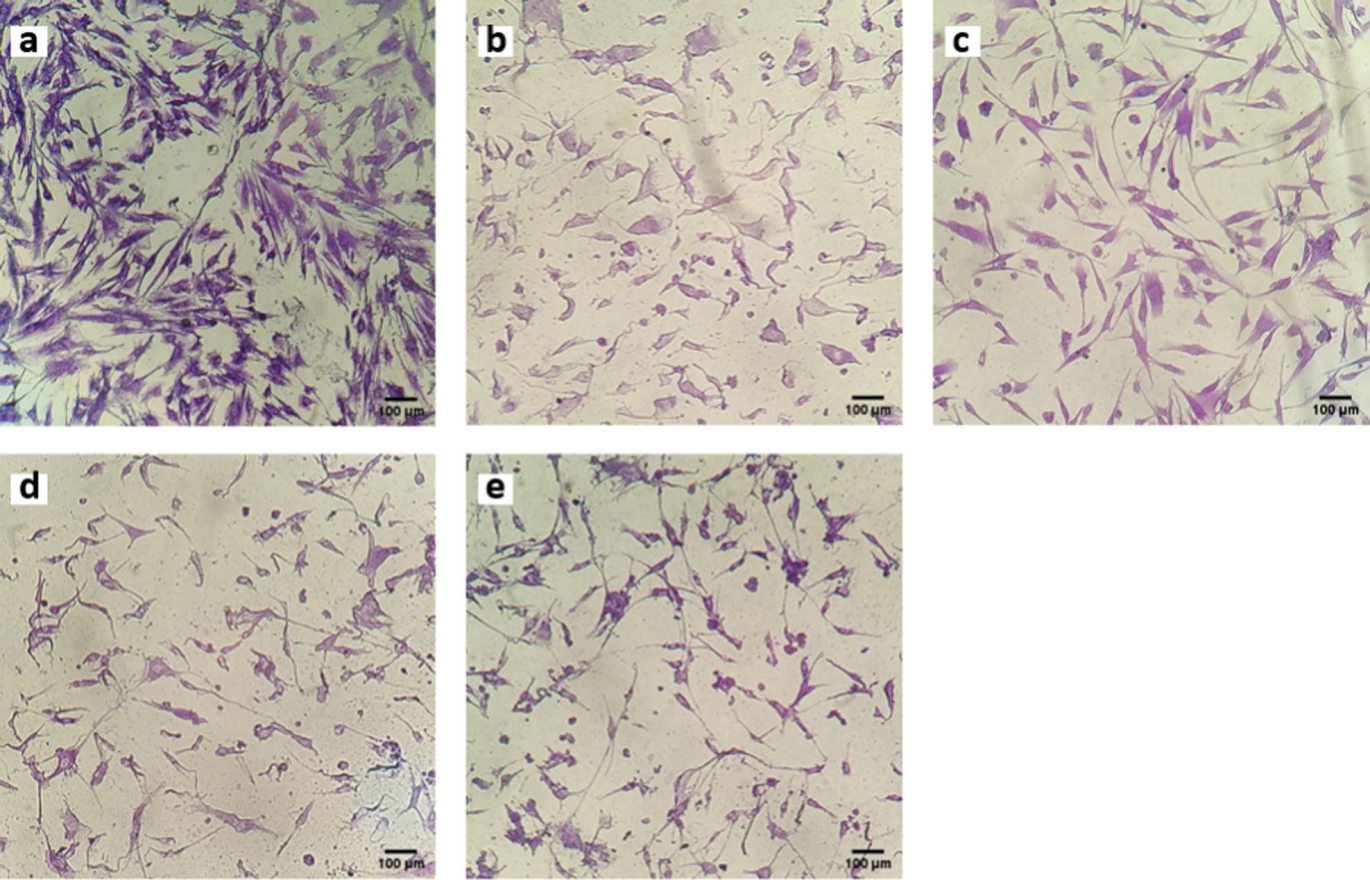
Microscopic images of Crystal Violet-stained fibroblasts in contact with extracts of the films for 24 h. **a** CWE, (**b**) PU, (**c**) PU-PEG 0.05, (**d**) PU-PEG 0.10, (**e**) PU-PEG 0.15

On the other hand, fibroblasts exhibit a similar behavior to that presented by osteoblasts when they were in contact with the extracts from the substrata (see Fig. 9). Thus, the morphology of fibroblasts in contact with PU, PU-PEG 0.10, and PU-PEG 0.15 extracts was irregular, but they presented a regular and spread morphology in contact with PU-PEG 0.05 extracts.

**Fig. 10:**
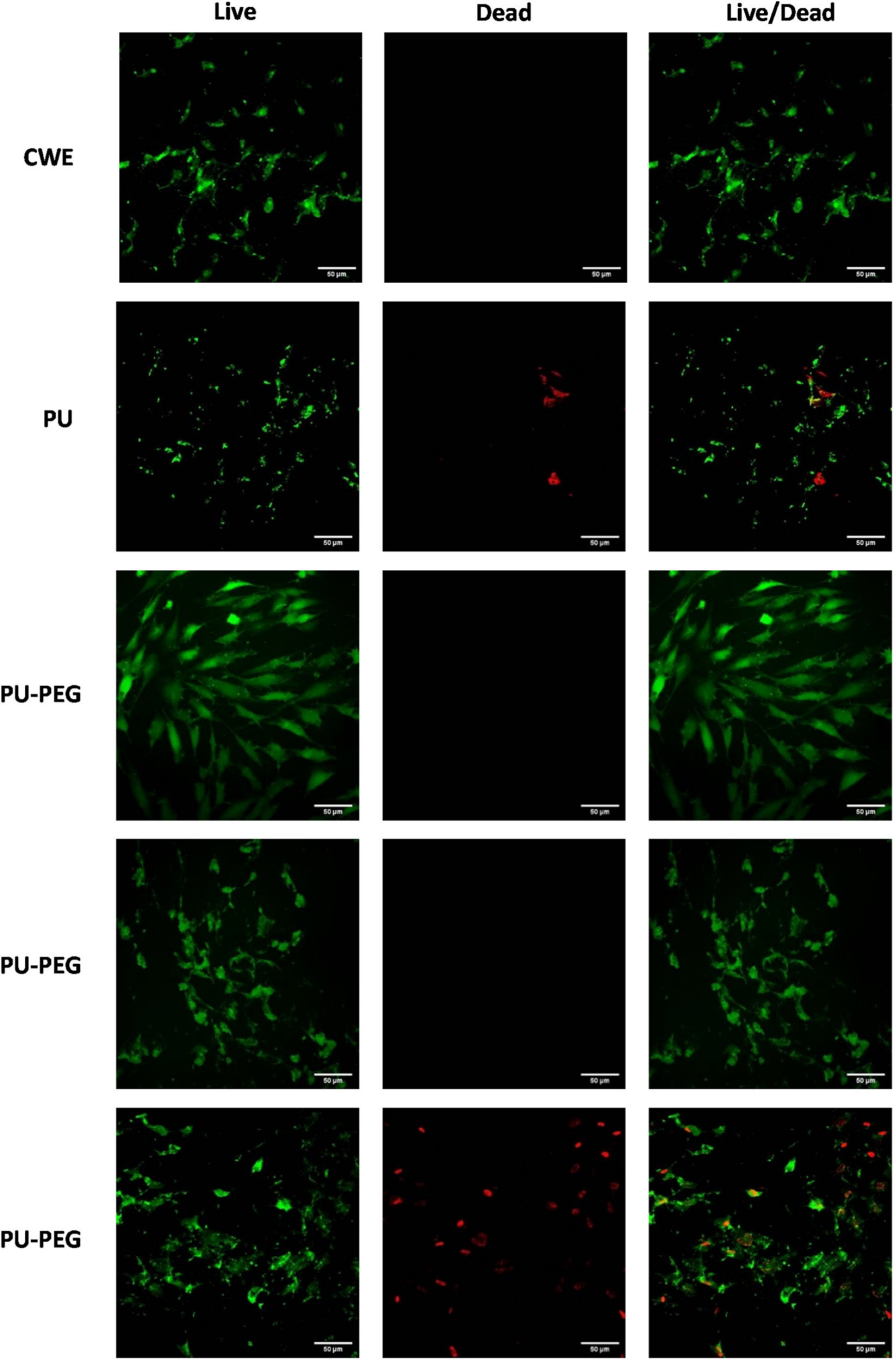
Confocal laser images of LIVE/DEAD staining of osteoblasts in contact with extracts of the films for 24 h

Osteoblast cells from Live/Dead staining assay exhibited the same behavior as the observed in crystal violet staining (see Fig. 10). Cells in contact with extracts from untreated PU showed an altered morphology and some cells have red fluorescence meaning that these cells are either dead or dying. Osteoblasts in contact with the extracts of PU-PEG 0.05 and PU-PEG 0.10 did not present dead cells; however, cells are observed not completely spread with PU-PEG 0.10 extracts, while the cells were fully spread with PU-PEG 0.05 extracts. Lastly, osteoblast cells in contact with PU-PEG 0.15 were dying as their nucleus displayed a red fluorescence meaning that they are damaged. Additionally, osteoblasts and fibroblasts were cultured on PU (control) and PU-PEG 0.05 substrates for 24 h and the microscopy images are included in Online Resource 3. As can be seen, no cells attached to the PU (control) substrates were observed, while osteoblasts and fibroblasts not only are adhered to the surface of PEG-modified-PU substrates, but also are spread over it.

## Discussion

PEG-grafted PU substrata with different PEG molar amounts were successfully synthesized and their surface properties and cell viability were evaluated. Thus, PEG grafting on the PU substrata was assessed through FTIR spectroscopy analysis. Surface modification reaction starts by grafting isocyanate groups onto PU surface; the secondary amine of the urethane linkage belonging to substrate reacts with one isocyanate group of the HMDI (leaving available an isocyanate group, which subsequently could react with a terminal hydroxyl group of the PEG in the second stage). The incorporation of the isocyanate groups onto substrata was confirmed by the presence of the characteristic band of this group at 2260 cm^−1^ in the FTIR spectrum of PU after the first stage of the reaction (please see the FTIR spectrum displayed in Online Resource 4). These isocyanate groups reacted completely during the second stage of the reaction as the band at 2260 cm^−1^ was not detected in the FTIR spectra of the PEG-grafted PU. Moreover, the changes observed in the FTIR spectra of the grafted PU films, i.e., that spectra were similar to that exhibited by PEG, together with an increase in the intensity of the band at 1342 cm^−1^, confirms that the reaction took place and that the amount of PEG in the substrata is higher as the PEG concentration used during the grafting reaction increase.

In addition, as the grafting reaction yields allophanate groups, an increase in these groups should occur. In this regard, it should be mentioned that the peaks related to allophanate structures reported in the literature vary depending on the molecular structure of the compounds; for instance, Ramakrishna et al. [36] reported allophanate peaks in the 1680–1620 cm^−1^ wave number range, while Zimmerer et al. [34] described two bands at 1714 and 1662 cm^−1^ associated with the carbonyls of the allophanates derived from N,N′-diisopropyl-N-carboxy-4-[1-(4-hydroxy-phenyl)-1-methyl-ethyl]-phenyl urea. These last peaks can be related to the bands A and C above mentioned, thus confirming the presence of allophanates in the PU; furthermore, the increase in the ratio C/(A + B) with the grafting reaction confirms that HMDI is reacting with the urethane bonds and more allophanate groups are being created with the PEG grafting density. It is important to note that, as the allophanate moiety has two carbonyls but only one secondary amine that can form hydrogen-bonds, therefore the number of free carbonyls is increased.

It has been reported that allophanate bonds have lower thermal stability than urethane bonds [37]; thus the formation of these groups can lead to that thermal stability of PU substrata were reduced; besides a fewer number of hydrogen-bonded carbonyls groups also could contribute to the reduction of thermal stability [38]. Here, it is worthy of mention that, due to the PU films swelling in toluene, the reagents are able to penetrate inside the films, so that grafting was carried out not only on the surface of substrata but also inside them modifying the bulk of the substrata, hence the impact on the thermal properties is appreciable. It is interesting to note that the lowering in the onset degradation temperature of the hard segment follows an inverse trend from the relation between free and hydrogen-bonded carbonyls (A/B) in Table 1; this corroborates that the decrease of the thermal stability is due to the reduction in the proportion of hydrogen-bonded carbonyls, as well as the presence of allophanate linkages.

Results obtained from SEM and AFM analysis revealed that PEG chains partially covered the surface of PU substrata, modifying their topology. This covering led to that the hydrophilicity of the substrata increased although their non-polar character also increased as evidence the contact angles values with diiodomethane and the non-polar component of their surface free energy. This behavior is due to the amphiphilic nature of PEG which has non-polar domains (–CH_2_–CH_2_–) [39], and the oxygens of the ether linkages that acts mainly as basic polar domains [40, 41]; which also led to an increase in the basic component of the grafted substrata. Besides this, the small increment in the acid component of PU-PEG 0.15 sample could be related to the presence of a higher OH groups from PEG chains, confirmed by the increment in the band at 1342 cm^−1^ in relation to the C–O band at 1111–1096 cm^−1^ as the PEG increases. Hydroxyl groups can act as electron acceptors which increase the affinity of the films to acid molecules [41], but as the OH are only present as end groups of PEG chains, their number is smaller than ether groups so their effect in the polar component is minor.

Biological characterization showed that the viability of osteoblast cells increased respect to the control (CWE) in all samples; this behavior was also detected when the osteoblasts viability of grafted samples was compared to that obtained for the non-grafted PU. In contrast, the fibroblast viability remained constant in all cases. This behavior could be attributed to the presence of PEG residues in the film extracts. It has been reported that the incorporation of PEG in copolymers improve the proliferation and spreading of osteoblasts on these materials [42–44]; for example, Piotrowski et al. [45] reported a higher osteoblasts viability in culture medium containing PEG-grafted fullerenes, attributing it to PEG biocompatibility and to the terminal OH groups. Our results concerning the osteoblasts viability test are in agree with this study; but, the attribution of the presence of OH groups from PEG on the cell viability would be inconclusive.

Some reports have also revealed that an alkaline pH in the osteoblast cellular medium increases both their viability and activity [46, 47]. As our results show above, the basic component of PU substrata increases with the amount of PEG grafted; thus, PEG could be raising the pH of the extracts. In order to prove this hypothesis, additional experiments were carried out to demonstrate that osteoblast viability increase with increasing the pH of the extracts. For this, PEG was added to the cell culture medium, and it was found that pH increased with the amount of PEG added (Online Resource 5). It has been reported that the ether groups of PEG can interact with carbon dioxide (CO_2_) molecules and capture them due to the Lewis acid-base interaction between CO_2_ and the electron donor ether groups (–O–) [48–51]. It should be remembered that DMEM medium requires the addition of sodium bicarbonate (NaHCO_3_) as a buffer to regulate the pH of the culture medium in the range 7.2–7.6 in incubated under a 5% CO_2_ atmosphere. Thus, the increase in the pH of the extracts could be explained by the interaction of CO_2_ molecules with PEG, instead of with NaHCO_3_, which could lead to an alkalization of the extracts and thus increase the viability of the osteoblasts. As PEG grafting density increases, more CO_2_ molecules could be captured by the higher amount of PEG, which would lead to the further increase in the pH of the extracts and higher viability of osteoblasts exhibited at the higher PEG grafting density.

It is important to note that osteoblasts play a role in the acid-base homeostasis, using bone as an alkaline buffer reserve [46], which explains the increased activity of these cells at an alkaline pH. On the other hand, fibroblasts do not have such a role in pH homeostasis; therefore, fibroblasts activity is not increased in alkaline pH. These difference between osteoblasts and fibroblasts viability, as well as the increase in the pH of the culture medium when PEG was added to it, allow us to confirm the suggested hypothesis which states that non-grafted PEG chains onto PU substrata can be released into the cell culture medium increasing the pH and thus increase the viability of osteoblasts. Despite this, it should be mentioned that although the viability of osteoblasts and fibroblast was not compromised by modified-PU extracts, the cells only showed a healthy morphology at low PEG amount (PU-PEG 0.05); at higher concentrations, the cell morphology is slightly altered.

## Conclusions

Results obtained in this work indicate that PEG was successfully grafted onto PU substrata. In this regard, FTIR and TGA results confirm that the grafting reaction take place in the urethane groups of the hard segment of PU trough the formation of allophanate linkages. It was also found that PEG chains modified the topography of the PU films by the yielding of a covering on their surface, which explain the higher hydrophilicity and surface free energy.

PEG grafting increased osteoblast cells viability, being higher as PEG amount is increased; which confirms that the modification of PU by the grafting of a more biocompatible and hydrophilic polymer like PEG is beneficial for biomedical applications. Despite this, the release of PEG in the media could be toxic at high grafting densities as the osteoblasts and fibroblasts only showed a healthy morphology with the lower grafting density of PEG (PU-PEG 0.05). Results also allow us confirm that PEG can increase the viability of osteoblasts due to the increase in the pH of the culture medium. Finally, it was demonstrated that surface properties such as surface energy play a key role in the viability of some cell types and that these features can be modified and may be tuned by the PEG grafting density. Alterations in cell morphology at high concentrations of PEG and the increase in the pH of the cell culture medium should be further analyzed.

## Supplementary Information

Supplementary Materials
