# Supplementary material for: Effect of PEG grafting density on surface properties of polyurethane substrata and the viability of osteoblast and fibroblast cells
Source: J Mater Sci Mater Med. 2022 May 18;33(6):45. doi: 10.1007/s10856-022-06668-1 (PMC9117377; doi:10.1007/s10856-022-06668-1)
Supplement: Supplementary file 1 — Supplementary Materials [file 10856_2022_6668_MOESM1_ESM.docx]

**Supplementary material**

Figure S1. TGA curves of the PU, PEG and grafted PU films. Thermal stability of the PU films is decreased with the grafting of PEG due to the formation of allophanate linkages.

Figure S2. DTGA curves of PU, PEG and grafted PU films. The onset degradation temperature is decreased and the form of the first peak associated to the hard segment degradation is modified for the grafted PU substrates due to the presence of allophanate groups.


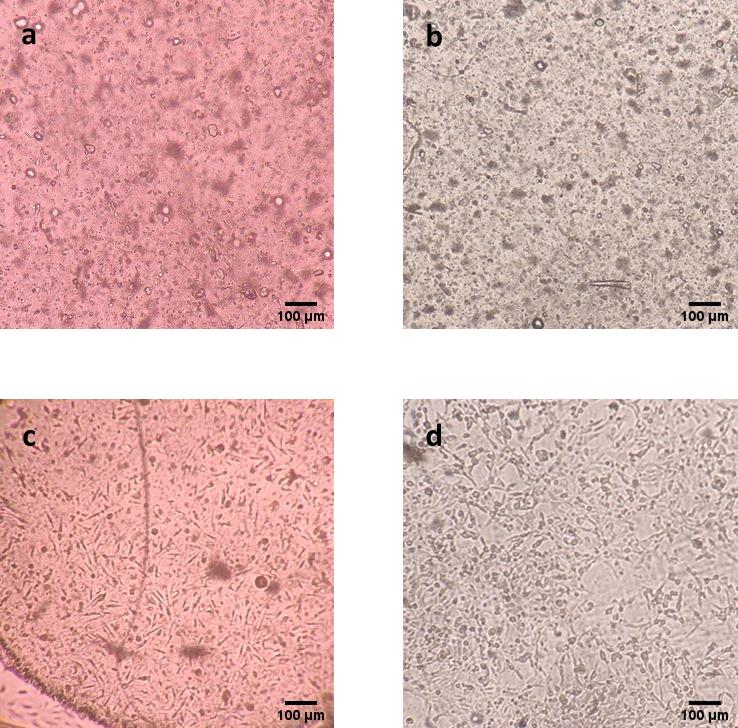


Figure S3. Fibroblasts and osteoblasts cultured on substrata for 24 h. a: fibroblasts/PU; b: osteoblasts/PU; c: fibroblasts/PU-PEG 0.05; d: osteoblasts/PU-PEG 0.05. No cells attached on PU, while both fibroblasts and osteoblasts attached and spread on PU-PEG 0.05.

Figure S4. FTIR-ATR spectra of HMDI grafted onto PU after the first stage of the grafting reaction. A very intense band at 2260 cm^-1^ indicates the presence of isocyanate groups.

| **Sample** | **pH Value** |
| --- | --- |
| DMEM | 8.5 |
| DMEM 0.5 μmol | 8.7 |
| DMEM 3.5 μmol | 8.8 |
| DMEM 9.5 μmol | 8.9 |
| DMEM 17.5 μmol | 9.0 |

Table S5. Values of pH measured from culture medium (DMEM) containing PEG. Culture medium without PEG was used as positive control (PC). pH increasing as PEG amount increased.
